# Importance of fatty acid binding proteins in cellular function and organismal metabolism

**DOI:** 10.1111/jcmm.17703

**Published:** 2023-03-06

**Authors:** Luis B. Agellon

**Affiliations:** ^1^ School of Human Nutrition McGill University Ste. Anne de Bellevue Quebec Canada

**Keywords:** Fabp, gene expression, metabolism, modulator, sensor, transporter

## Abstract

Fatty acid binding proteins (Fabps) are small soluble proteins that are abundant in the cytosol. These proteins are known to bind a myriad of small hydrophobic molecules and have been postulated to serve a variety of roles, yet their precise functions have remained an enigma over half a century of study. Here, we consider recent findings, along with the cumulative findings contributed by many laboratories working on Fabps over the last half century, to synthesize a new outlook for what functions Fabps serve in cells and organisms. Collectively, the findings illustrate that Fabps function as versatile multi‐purpose devices serving as sensors, conveyors and modulators to enable cells to detect and handle a specific class of metabolites, and to adjust their metabolic capacity and efficiency.

## 1 INTRODUCTION

**FIGURE 1:**
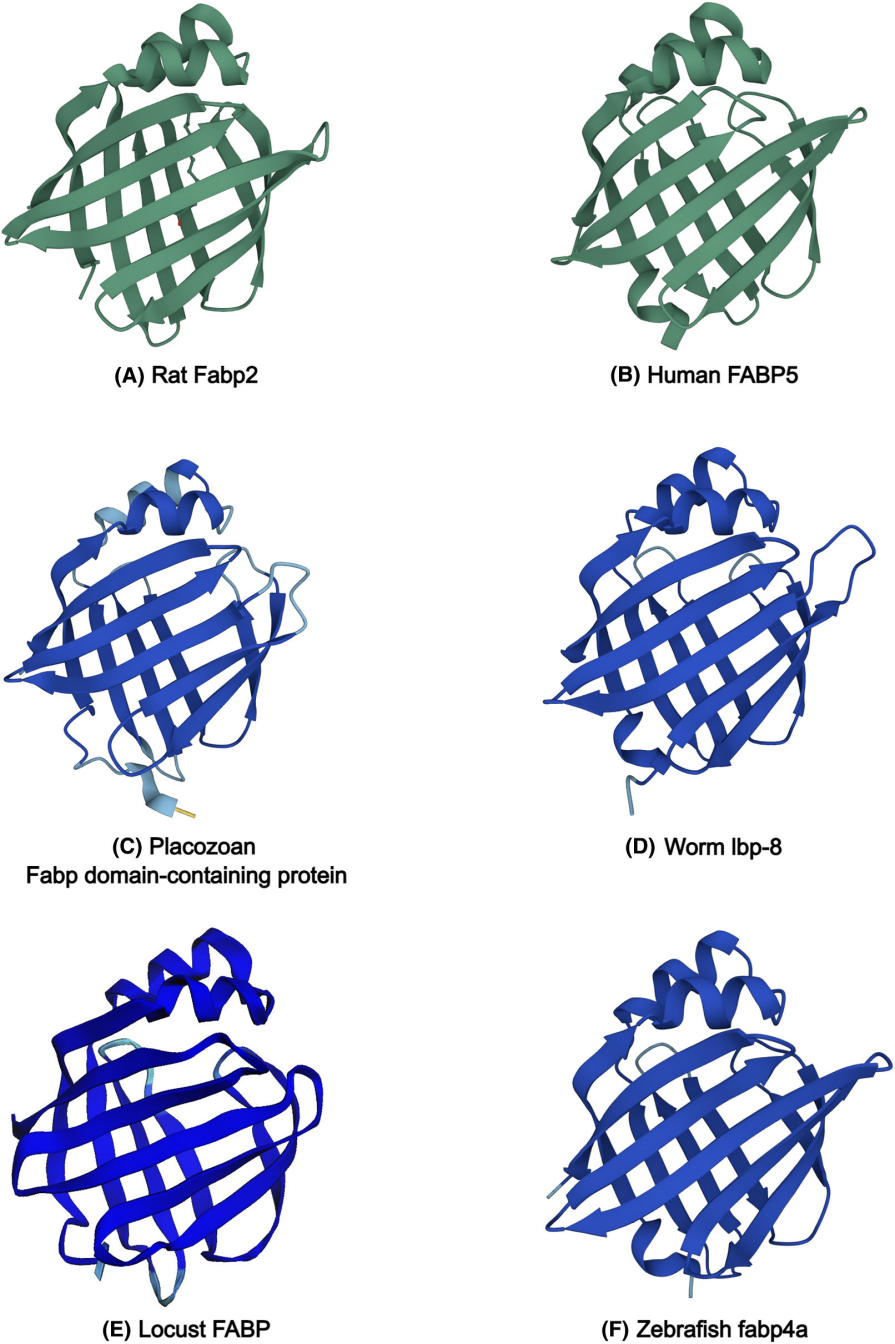
Structures of Fabps from different organisms determined experimentally (A, B) or predicted by AlphaFold^43^ (https://alphafold.ebi.ac.uk/) (C–F). (A) Rat Fabp2 with bound ligand, determined by x‐ray crystallography (RCSB PDB 2IFB). (B) Human FABP5, determined by NMR spectroscopy (RCSB PDB 1JJJ). (C) *Trichoplax adhaerens* FABP domain‐containing protein (AlphaFold entry B3S4H2). (D) *Caenorhabditis elegans* lbp‐8 (AlphaFold entry O02324). (E) *Schistocerca gregaria* Fabp structure predicted from nucleotide sequence (NCBI accession XP_049839815.1) using AlphaFold2 via Colaboratory (https://colab.research.google.com/). (F) *Danio rerio* fabp4a (AlphaFold entry Q66I80).

Fabps belong to a large set of lipid binding proteins known as the calycin superfamily that share common structural motifs and bind small hydrophobic molecules.^1^ The Fabps, along with structurally related cellular retinol binding proteins,^2^ are distinct from other members of the calycin superfamily in that they share remarkable conservation of protein three‐dimensional structure (Figure 1) despite low primary sequence identity.^3^ Over the last half century, research on Fabps has garnered thousands of research papers, including hundreds of review articles.

It is well established that members of the Fabp family are cytosolic proteins comprised of a single polypeptide chain, and that they bind a wide assortment of small hydrophobic molecules such as fatty acids and their metabolites, signalling molecules and synthetic compounds.^4^, ^5^, ^6^, ^7^, ^8^, ^9^, ^10^ Fabp orthologs are represented in a wide variety of organisms, from placozoans to chordates (Figure 1).^11^, ^12^ The apparent conservation of Fabp proteins through evolution and their characteristic high abundance in cells suggest that these proteins are important for survival. In mammals, it is not unusual to find more than one Fabp paralog in one organ, or even in one cell‐type.^13^, ^14^ Indeed, many roles for Fabps have been suggested, such as storage reservoir, buffer, intracellular lipid chaperones, as well as regulators of gene expression and metabolic function. Strikingly however, genetic ablation of specific Fabp genes do not seem to cause lethality nor detrimental phenotypes, although their absence has been seen to cause some metabolic perturbations that are evident at the organismal level.^14^, ^15^, ^16^, ^17^ In some cases, loss of Fabps modify gene expression programs in a sex‐dependent manner.^18^ Another notable feature of Fabps is the correlation of their increased abundance in individuals with particular diseases or pathological conditions. For this reason, it has been suggested that the concentration of certain Fabps in the blood, urine or tissue samples are useful diagnostic or prognostic markers.^19^, ^20^, ^21^, ^22^ Yet, the exact relevance of these proteins in cellular and organismal existence remains enigmatic as the precise functions that Fabps serve in cells and organisms has proven to be elusive through 50 years of study.

## 2 DISCOVERY OF FABPS

In 1972, Ockner et al.^23^ reported obtaining a small protein from rat intestinal extracts and named it “fatty acid binding protein” as it showed tight association with a radiolabelled long‐chain fatty acid. This fatty acid binding activity was also evident with similarly sized proteins obtained from other tissues,^23^ including a protein referred to as the Z protein.^24^ The Z protein is a component of one (Fraction Z) of two protein fractions obtained by size exclusion gel filtration chromatography of liver supernatants that showed binding activity to organic anions.^24^ Today, a group of similarly sized proteins present in many tissues and displaying the same propensity for binding fatty acids along with other comparable biochemical properties comprise a family of proteins referred to as the fatty acid binding protein family.^3^, ^23^, ^24^, ^25^

It is established that there are ten distinct types of Fabps in humans (Table 1), including Fabp12 which is the latest member to be discovered.^26^ Some types have widespread tissue distribution while others are restricted to one or a few tissues (Table S1). Selected examples include Fabp5 which appears to be the most widely distributed paralog of the Fabps in humans (Table S1), Fabp6 which resides in intestinal and ovarian tissues^27^ and Fabp8 (legacy name: PMP2) which is restricted to nervous tissue. The existence of multiple types of Fabps suggests that these proteins play different roles.

**TABLE 1:** The human FABP gene family.

Systematic name	Legacy name	Chromosomal location^a^
*FABP1*	Liver fatty acid binding protein (L‐FABP)	2p11.2
*FABP2*	Intestinal fatty acid binding protein (I‐FABP)	4q26
*FABP3*	Heart fatty acid binding protein (H‐FABP)	1p35.2
*FABP4*	Adipocyte lipid binding protein (ALBP)	8q21.13
*FABP5*	Epidermal fatty acid binding protein (E‐FABP)	8q21.13
*FABP6*	Gastrotropin	5q33.3
*FABP7*	Brain fatty acid binding protein (B‐FABP)	6q22.31
*FABP8*	Peripheral myelin protein 2 (PMP2)	8q21.13
*FABP9*	Testicular lipid binding protein (TLBP)	8q21.13
*FABP12*	‐	8q21.13

^a^ Chromosomal locations were obtained from http://www.genome.ucsc.edu/, accessed 2022–10.

Substantial knowledge has been gained regarding the ligand preferences of each Fabp type. All have in common the tendency to bind small hydrophobic compounds of natural and synthetic origins.^4^, ^5^, ^6^, ^9^, ^10^ Many Fabp types overlap in their ligand preferences, which provides cells that express multiple Fabp types with redundant capability. It is of interest to mention that some Fabps have stricter ligand preferences, such as bile acids for Fabp6 or phosphoinositides for Fabp8.^6^, ^10^ Since novel members of the Fabp multi‐gene family seem to appear in other organisms such as worms^28^ and fish^29^ (e.g. Figures 1D,F), a question that follows is why novel types of Fabps arise. From an evolutionary standpoint, a possible evolutionary driver is the need for Fabps that have different ligand specificities and/or specializations.

## 3 FABP STRUCTURE AND PUTATIVE FUNCTIONS

### 3.1 Gene structure

All the expressed Fabp genes in the human genome share a similar genomic organization but their primary structures, particularly in the gene promoter regions, show substantial diversity indicating distinct mechanisms regulating their expression, including their characteristic patterns of tissue‐specific expression. In the human genome, several Fabp genes exist in a cluster on human chromosome 8 (Table 1), which is thought to have been generated by gene duplication followed by diversification, while the remaining family members are distributed to other autosomes.^3^, ^26^ Fabp genes appear to be ancient, as versions can be found from placozoans to chordates.^11^, ^12^ Fabp multigene families are found in markedly different animals, such as mammals, nematodes,^28^ teleosts^29^ among others. The primary structures of Fabp gene paralogs show greater divergence as compared to Fabp gene orthologs. In some organisms, new versions of Fabps likely arose after divergence from the common evolutionary trunk, as suggested by the primary structures of zebrafish *fabp* gene family members.^29^ The *Drosophila melanogaster* genome contains only one copy of the Fabp gene,^30^ however splice variants have been found and it has been suggested that alternative splicing of the Fabp transcript might be another mechanism for generating Fabp variants from the single Fabp gene.^11^ In humans, associations between allelic variants of several Fabp genes with distinct metabolic profiles are known to exist. To illustrate, it was reported that the A54T variant of the *FABP2* gene is associated with obesity and insulin resistance^31^ while the *FABP1* T94A and *FABP6* T79M variants are each associated with yet different metabolic profiles.^32^, ^33^ In the case of the *FABP2* A54T variant, the many studies that have been carried out indicate that the phenotype of this variant is not consistent among different populations; for example, one study done on a Japanese cohort did not find an association of the *FABP2* A54T with obesity nor insulin resistance.^34^ It has been suggested that there are likely other factors involved in determining the phenotypic outcome of this specific gene variant.^35^ Indeed, targeted inactivation of the *Fabp2* gene in mice results in a sex‐dimorphic phenotype.^36^ On the other hand, the inactivation of the *Fabp1* gene in mice of different genetic backgrounds manifests divergent phenotypes.^37^ It seems paradoxical that the evolutionary history of the Fabps suggests the existence of selective pressures driving the emergence and preservation of multiple types of Fabp in one cell‐type or organism, yet these proteins seem to be dispensable, and their loss do not result in a consistent phenotype. The different types of Fabp proteins are likely serving a variety of important purposes but the consequences of their actions depend on their environments.

### 3.2 Protein structure

The first three‐dimensional structure of an Fabp was solved by x‐ray crystallography (Fabp2^38^ shown in Figure 1A) and revealed that the protein consists of ten β strands that form two β sheets arranged to create a barrel structure. The outer surface of the barrel is comprised of hydrophilic sidechains, albeit non‐identical among different Fabp types, while the interior surface of the barrel is lined with hydrophobic sidechains that are also variable among the Fabps. A helix–loop–helix motif is connected to one end of the barrel to form a lid at the portal region of the protein. The structure was confirmed using nuclear magnetic resonance spectroscopy (NMR) (e.g. FABP5^39^ shown in Figure 1B). The structure determined by NMR of unliganded Fabp2 in solution also revealed that the α helices exhibit a greater degree of structural “disorder” (the “open” configuration) but become more rigid (the “closed” configuration) upon binding of ligand.^40^, ^41^, ^42^ As the tertiary structures of different Fabps became known, an attribute of Fabp proteins that stands out is the astonishing similarity in protein fold despite low sequence identity. Figure 1 illustrates this feature in the Fabp structures of animals from various phyla that have been determined experimentally,^38^, ^39^ or predicted from primary structures using AlphaFold.^43^ Another important feature observed is the subtle change in the overall conformation of Fabps upon ligand binding. In general, the induced changes in overall structural conformation induced by the binding of ligand appears to be characteristic depending on both the type of Fabp and the specific ligand.^42^, ^44^, ^45^

Not surprisingly, some genetic polymorphisms make distinct contributions to the structure of Fabps and, depending on their locations, influence the ability of the variants to interact with their binding partners or to membranes. It is interesting to note that the polymorphic amino acid residues of FABP1 T94A, FABP2 A54T, FABP6 T79M, and FABP8 F57A are situated in the portal region of these Fabps.^46^, ^47^, ^48^, ^49^ Structural changes imparted by the altered residues on the biochemical properties of these proteins are no doubt involved in expressing their phenotypes, although how these are accomplished is not exactly known. Amino acid residues in the two α helices that form the lid as well as those in the hinge and portal regions have been seen to be important in ligand selection, binding/release, and interaction with membranes but the nature and sequence of changes that results in the alteration of the metabolic programs have not yet been fully worked out. Although some genetic variants contribute little change in ligand binding affinity, it is possible that the variant amino acids alter Fabp structure sufficiently to influence other Fabp interactions.

It was found that some Fabp types undergo a variety of posttranslational modifications and are seen to occur in several cell‐types under specific metabolic settings.^50^, ^51^, ^52^, ^53^ The impact of posttranslational modifications on protein structure and action of Fabps has received far less attention compared to Fabp‐ligand interactions. Dynamic posttranslational modifications are commonly used by cells to regulate the capacity of the modified proteins to carry out their catalytic activity or to interact with their substrates and other binding partners. Some examples of posttranslational modification of Fabps include phosphorylation of Fabp3 and Fabp4,^51^, ^52^, ^53^ 4‐Hydroxynonenal modification of Fabp1, Fabp4, and Fabp5,^54^, ^55^, ^56^ and ubiquitination of Fabp1.^55^ Some of these modifications have been shown to alter the interaction of Fabps not only with their ligands but also with other protein interaction partners. Since Fabps are normally present in high abundance in the cell cytoplasm, it is likely that only a small fraction of the cytoplasmic pool of Fabps that becomes modified is sufficient to influence their interactions with their binding partners and cause changes in cellular metabolic activity. A concept that is suggested by currently available data is that the structure of Fabp is analogous to a flexible sac‐like container whose external features can be remodelled by the ligands and/or posttranslational modifications they carry.

### 3.3 Putative functions

As already mentioned, the finding that Fabps are present in high abundance in the tissues of many organisms inspired the notion that these proteins served the purpose of buffering, storing and/or transporting their ligands. Fabps are particularly abundant in the cells of organs that actively handle or metabolize lipids, and cells possess molecular mechanisms to increase or decrease the abundance of Fabps in response to changes in the amount of available ligand as well as the extent of cellular metabolic activity. It could be rationalized that Fabp paralogs evolved so that cells could handle specific types of lipids. In vitro studies employing purified rat Fabp1 and Fabp2 proteins and model membranes showed that Fabps can facilitate the transfer of their ligands between membranes, but the mechanism of transfer is not the same for these Fabps.^57^ Furthermore, the specialization of these paralogous proteins is maintained by their respective orthologs.^57^ Also noteworthy was the finding that certain amino acid residues in the portal region of Fabps play an important role in specifying the biochemical properties of Fabps, and explains why the FABP2 A54T variant has a reduced capacity for fatty acid transfer between membranes compared to the prototype FABP2 A54.^47^ Later studies further demonstrated that the α helical domains of Fabp1 and Fabp2 account for the difference in their mechanism of fatty acid transport.^58^

The biochemical studies that employed purified Fabps and synthetic membranes also illustrated that Fabps were, by themselves, responsible for the transport of their cargoes, since no other proteins were present in the assay system. However, later studies found that some Fabp types can and do make direct interactions with membrane‐bound transporters to acquire their cargoes, such as Fabp3 and Fabp4 with CD36 or Fabp6 with apical bile salt transporter.^59^, ^60^, ^61^ Additionally, some Fabps were observed to translocate into the nucleus and interact with specific transcription factors belonging to the nuclear hormone receptor family to modulate their activities.^61^, ^62^, ^63^, ^64^, ^65^, ^66^, ^67^ It has been shown that some types of Fabps possess a cryptic non‐standard nuclear localization signal in their α helical domains which becomes readily unmasked upon binding of appropriate ligands.^64^, ^65^, ^67^ A similar motif is evident in the human cellular retinoic acid‐binding protein II, which delivers retinoic acid to retinoic acid receptors resident in the nucleus.^68^ Fabp‐nuclear receptor interactions are influenced by the type of ligand bound by Fabps, as revealed by ligands of Fabp1 and Fabp2,^69^ as well as amino acid substitutions in the portal regions, as demonstrated by the Fabp1 T94A variant.^44^, ^70^ Fabp5 has also been shown to interact with HIF‐1α, a transcription factor involved in the cellular response to hypoxia.^71^ Collectively, these observations have provided information on the mechanisms by which certain kinds of metabolites and other small lipophilic compounds can coordinately regulate the expression of genes that are involved in different metabolic pathways and cellular processes.

In addition to transcription factors, Fabps are also found to interact with other cellular proteins and influence the activities of their interacting partners; some examples include Fabp4 and Fabp5 interaction with hormone sensitive lipase,^72^ Fabp3 with the cytoplasmic tail of integrin α‐subunits,^73^ and recently, Fabp5 with the cytosolic tail of calnexin,^74^ as well as several Fabp types with comparative gene identification‐58 (Cgi‐58) protein.^75^ It is interesting to note that Fabp3 has also been referred to as a growth inhibitor whereas other Fabps, for example Fabp4, have been reported to stimulate cell proliferation.^76^, ^77^ The mechanism(s) by which Fabps influence cell growth is not clear. Fabps have even been found in tight association with very‐low density lipoprotein and chylomicron transport vesicles.^78^, ^79^ These observations illustrate the participation of Fabps in a variety of cellular processes.

The ubiquity of Fabps suggests that these proteins are integrated into the cellular machinery. One difficulty in ascribing a specific function to Fabps is their lack of any known enzymatic activity and therefore their actions, apart from binding and sequestration of their preferred ligands, is difficult to surveil. Considering the ability of the Fabps to interact with many cellular components, it is necessary to reconsider the functions contributed by Fabps in the regulation of gene expression and coordinating of cellular metabolism. It was originally anticipated that the phenotypes of genetically engineered mice carrying inactivated Fabp genes would shed insight into their specific functions. Unexpectedly however, engineered mice lacking one or more of Fabp types were viable, fertile, and displayed seemingly normal phenotypes although they manifested some sort of systemic metabolic dysregulation under metabolic challenge.^14^, ^15^, ^80^, ^81^ Mice lacking Fabp3 (legacy name: H‐Fabp) are viable but become exercise intolerant and more prone to cardiac disease.^80^, ^82^ Mice lacking Fabp5 (legacy name: E‐Fabp) exhibit a slight diminution of basal transepidermal water loss,^83^ but became resistant to experimental autoimmune encephalomyelitis (EAE) induction, a mouse model of inflammatory demyelinating disease.^84^ It also became apparent that sex is an important modifying parameter since males and females do not always show the same outcomes from Fabp deficiency.^15^, ^18^, ^36^, ^85^ Furthermore, Fabp1‐deficient mice of different genetic backgrounds do not show identical phenotypes, indicating that this specific Fabp type is not serving one dominant functional role.^37^ Thus, although Fabp gene inactivation did not explicitly reveal the fundamental functions of Fabps, these studies did hint that context is an important parameter in elaborating the phenotypes caused by Fabp gene inactivation.

## 4 RECENT DEVELOPMENTS

Studies reported in the last 5 years demonstrate that Fabps are intimately involved in many facets of cellular function. It is now well established that Fabps collectively have a sophisticated repertoire of ligands. Structural studies have provided a wealth of information on how Fabps interact with their ligands at the molecular level.^41^, ^42^, ^44^, ^45^ Whereas some types of Fabps have relaxed ligand selectivity, others are more selective. It was recently found that Fabp8 binds phosphoinositides and revealed the intimate involvement of Fabp8 in the asymmetric modelling of sphingomyelin in the inner and outer leaflets of cell membranes of rat hepatoma cells expressing recombinant Fabp8, and thus likely also in the cell membranes of Schwann cells where Fabp8 is normal present.^10^ One feature revealed by structural studies that is not well appreciated is the functional significance of the subtle but distinctive remodelling of Fabp tertiary conformation that occurs upon binding of specific ligands.^41^, ^42^, ^44^, ^45^ This feature appears to have been exploited by cells that express multiple types of Fabps to recognize specific molecules within a population of competent ligands in order to differentially regulate the efficiency of distinct metabolic pathways.

Conventional Fabp‐deficient mouse models failed to clearly and definitively elaborate the functions of Fabps although these animal models did reveal that context (e.g. sex, genetic background, nutrient status) is an important modifier of the phenotypic outcomes of Fabp gene inactivation.^14^, ^15^, ^37^, ^85^, ^86^ In regards to phenotypic differences observed between sexes and in mice with different genetic backgrounds, it is now known that the gene expression programs of males and females, or that of different mouse strains, are not identical.^18^, ^87^, ^88^ Alternative methods for inhibiting gene expression in wild‐type organisms are now available. For instance, the inability of cardiomyocytes to utilize fatty acids as a metabolic fuel due to the absence of Fabp3 was previously postulated to be the explanation for exercise intolerance manifested by mice lacking Fabp3.^80^, ^82^ The same outcome of muscle Fabp deficiency was demonstrated directly in wild‐type desert locust (*Schistocerca gregaria*) through the use of RNAi‐mediated inhibition of muscle Fabp gene expression and on‐demand induction of physical exertion via a flight reflex.^89^ Thus, systems that permit targeted and direct alteration of Fabp gene expression and Fabp protein abundance can be more informative in identifying cellular processes and pathways that are primarily dependent on the function of Fabps.

The increased expression of Fabp7 is associated with poor prognosis of survival for patients with glioblastoma, a highly aggressive form of brain cancer.^90^ Increased abundance of Fabp7 promotes cell migration in vitro, and Fabp7 is evident at the leading edges of infiltrative tumours.^91^ High numbers of mitochondria are also evident within invadopodia of invading cancer cells,^92^ including astrocytic tumours.^93^ More recently, high resolution imaging techniques showed that supplementation of the growth medium of cultured glioma cells with docosahexaenoic acid, a known Fabp7 ligand, causes the relocation of Fabp7 from the plasma membrane to mitochondria and the inhibition of cell migration.^91^ Another possibility is that a switch of fuel substrate, facilitated by efficient delivery of the Fabp7 ligand to mitochondria, is responsible for the inhibition of cancer cell migration. Notwithstanding changes in membrane lipid composition and topology, the change in Fabp7 protein conformation resulting from the binding of its substrates may alter the affinity of liganded Fabp7 to yet unidentified protein(s) or protein complex(es) on mitochondria to alter mitochondrial function.

The discovery that some Fabps interact with membrane‐bound transporters and nuclear receptors offered a framework for how abundant metabolites, such as fatty acids, can regulate gene expression to modify cellular metabolism. The physiological significance of the interaction of Fabps with other cellular proteins was more difficult to ascertain but more recent findings have provided insights into the importance of such interactions. For example, it was found that the interaction of Fabp5 with Mammalian SEC12 (also called prolactin regulatory element‐binding protein) enhances the budding of lipoproteins in vitro,^94^ and suggests that Fabp1 and Fabp2 carry out the same role in the biogenesis of lipoproteins in hepatocytes and enterocytes.^78^, ^79^ It may well be that Fabps, depending on the type, specific ligand bound and state of posttranslational modification, participate in modulating the activities of other cell proteins.

Another important insight into the versatility of Fabps came from the unanticipated common phenotype of two distinct mouse strains: one that is deficient of the cytosolic Fabp5 protein and the other that is deficient in calnexin.^95^ Fabp5 was initially found in epidermal cells and becomes elevated in psoriatic skin lesions.^20^, ^96^ Fabp5 is also found in endothelial cells^96^ and explains why this Fabp type is detectable in many organs including the brain. It has been shown that Fabp5 is required for the efficient uptake of docosahexanoic acid across the blood–brain barrier.^97^ As mentioned earlier, loss of Fabp5 conferred protection against EAE.^84^ Surprisingly, mice lacking calnexin are similarly resistant to EAE.^95^ Calnexin is an endoplasmic reticulum‐resident protein chaperone that is structurally related to another endoplasmic reticulum‐resident calcium binding protein known as calreticulin.^98^ However, unlike calreticulin which is a luminal endoplasmic reticulum protein, calnexin is comprised of an N‐terminal domain that is situated in the lumen of the endoplasmic reticulum, a transmembrane domain and a C‐terminal region that is exposed to the cytosol. Importantly, Fabp5 is capable of forming stable interactions with the cytosolic C‐tail domain of calnexin.^74^ A subsequent study demonstrated that the formation of the Fabp5/calnexin complex is necessary for restoring the sensitivity of mice to EAE.^99^ In fact, the specific loss of just the calnexin C‐tail domain is sufficient to impart resistance to EAE induction.^99^ Using both mice and a cell culture model of the blood–brain barrier, it was found that the ability of activated T‐cells to cross an endothelial cell layer is dependent on the stable interaction of Fabp5 with the cytosolic C‐tail domain of calnexin at the endoplasmic reticulum.^99^ The precise sequence of events and the identity of other players involved in the pathogenesis of EAE and whether the type of ligand bound to Fabp5 influence the formation of Fabp5/calnexin complex are not yet known. Although the Fabp5 binding site on the calnexin C‐tail domain has not yet been mapped, it is likely that their interaction interferes with the binding of other calnexin C‐tail interactors, such as SH3 domain GRB2 like endophilin interaction protein, SUMOylation E2 ligase, protein tyrosine phosphatase 1B, N‐myristoyltransferase, HIV protein negative regulatory factor and activity‐regulated cytoskeleton‐associated protein.^100^, ^101^, ^102^, ^103^, ^104^ As well, Fabp5 has been observed to translocate to the cell nucleus where it has the capacity to regulate the expression of genes involved in multiple pathways.^63^, ^71^ It is possible that the unique conformations of Fabp5 induced by binding of specific types of ligands offers selectivity of pathways to be modulated by the Fabp5‐ligand complex. Nevertheless, the studies on the Fabp5/calnexin complex illustrate that changes in cellular function and pathophysiology can be directly linked to the interaction of a specific Fabp type with other cellular proteins.

## 5 A COHERENT WORKING MODEL OF FABP FUNCTION

**FIGURE 2:**
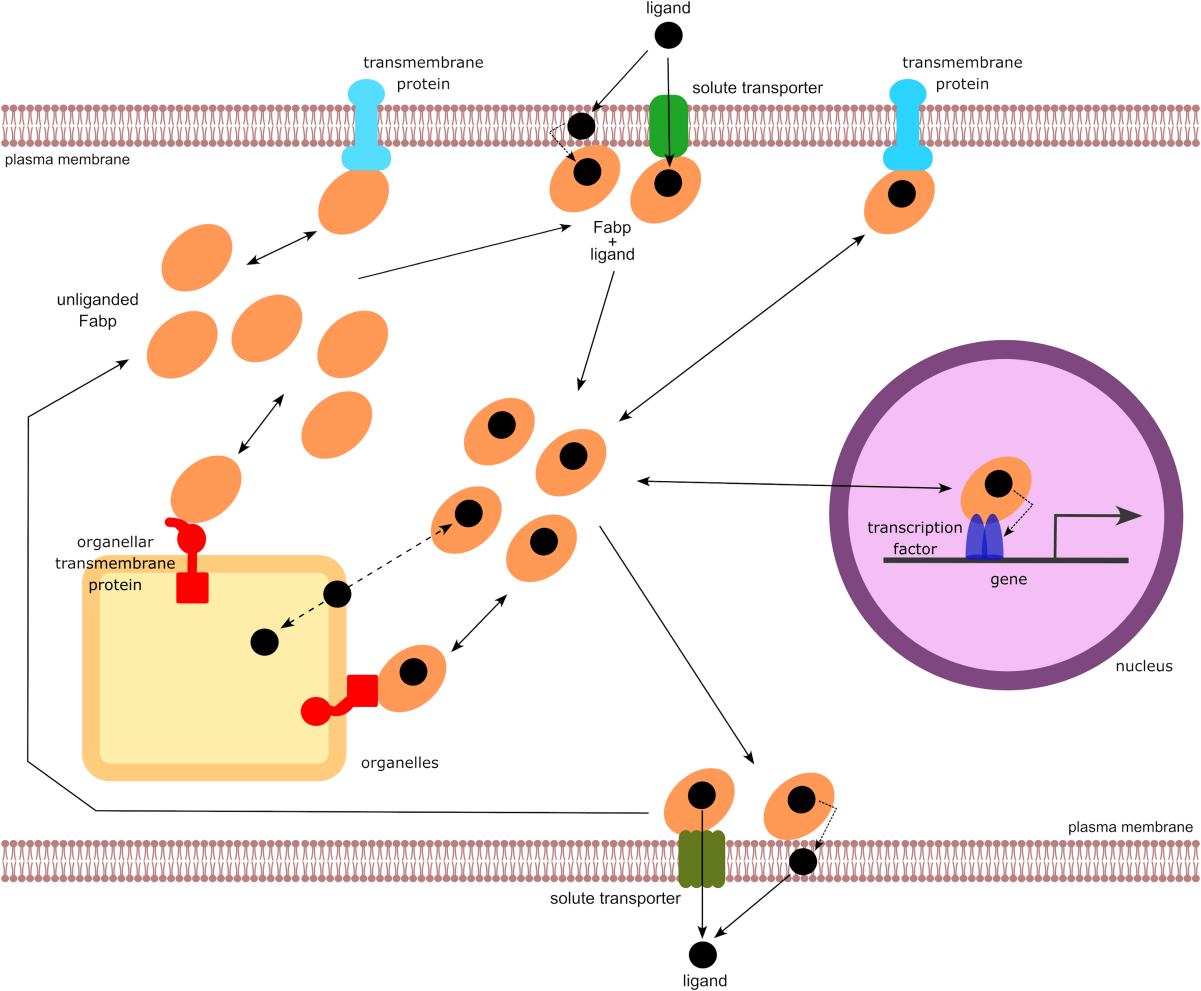
Model of Fabp function. Fabps function as sensors, conveyors and modulators of cellular function. Small hydrophobic molecules (e.g. fatty acids, bile acids) that serve as Fabp ligands (black circle) enter cells via solute transporters (represented in green), or partition into the plasma membrane, are taken up by Fabps (represented in light brown). Each Fabp type has distinct ligand preferences as well as preferred protein binding partners (e.g. represented in orange and cyan in the figure) with different affinities for unliganded and liganded Fabps. Liganded Fabps transport their cargos to their sites of utilization. These include the nucleus (represented in purple) and other organelles (represented in beige). In the nucleus, Fabps interact with a variety of transcription factors (represented in blue), including but not limited to nuclear receptors which accept their cargos, to regulate the expression of their target genes. Fabp ligands conveyed into the nucleus may leave the nucleus via Fabps. In other organelles, both unliganded and liganded Fabps can interact with the cytosolic domains of membrane‐bound transmembrane proteins to modulate their activities (organellar transmembrane proteins represented in orange; plasma membrane transmembrane proteins represented in cyan). Fabp ligands delivered to organelles can be metabolized (e.g. fatty acids converted to triacylglycerols for storage or oxidized for energy). Alternatively, fatty acids released during hydrolysis of triacylglycerols, or other fatty acid esters, can be captured by Fabps and conveyed to other sites of utilization. Fabps may offload their cargos to various membrane systems (plasma and organellar membranes) by desorption, or via membrane‐bound transporters. Cellular export of Fabp ligands can be achieved by interaction of the liganded Fabp with plasma membrane‐localized solute exporters (e.g. represented in green at the bottom of figure).

Available data clearly show that Fabps are entrenched in many cellular processes. The parsimonious explanation for the existence of Fabps is that cells employ these proteins as versatile multi‐purpose molecular devices (Figure 2). As sensors, Fabps sense the intracellular presence of specific molecules. As conveyors, these proteins transport their cargoes to sites of ligand utilization or carry information about their cargoes to other components of signalling pathways in the cell. As modulators, they adjust the effective activities of other cellular proteins that serve as part of the cellular machinery. The high concentration of Fabps in the cytoplasm and their overlapping ligand preferences ensures that there is sufficient capacity to accommodate high fluxes of their ligands. The refined selectivity and greater affinity of some Fabp types for certain kinds of ligands enables cells to recognize molecules that serve a special purpose, such as a regulatory role. These facets of Fabp function are a consequence of the 3D structure shared by all Fabp types and the multiplicity of their surface topologies given by their diverse primary structures. The binding of ligands by Fabps modifies their 3D conformation in a manner that is characteristic to the specific ligand but in ways that differ for each type of Fabp. Some types of Fabps interact with certain proteins and the degree of interaction is dictated by the presence and type of bound ligand. In addition, posttranslational modification of Fabps by dynamic covalent attachment of prosthetic groups add further complexity to Fabp protein conformation which alter the affinity of the modified Fabp towards its ligands and in turn promote or inhibit Fabp interactions with other interaction partners thereby modulating their activities. Thus, while Fabps are not strictly necessary for cellular viability, their existence offer cells the enhanced capacity to sense the presence of their ligands and to adjust the efficiency of the cellular machinery accordingly. The outcome of Fabp action is dictated by its immediate surroundings which is comprised of the spectrum of ligands, metabolites and proteins present in the cell.

## 6 SUMMARY

The concept that emerges from the sum of knowledge generated by five decades of study is that Fabps arose and evolved to be adaptable multipurpose cellular tools. The elegant architecture of the Fabp protein allows for tailoring of structure to serve specific purposes. Fabps participate in a myriad of cell processes, but they do not necessarily act as master regulators of cellular functions. Rather, Fabps are modular components that serve important roles in many metabolic and regulatory pathways. These proteins are not essential for cell viability per se, but they do contribute substantially towards the adjustment of cellular function. However, as the outcome of Fabp action is context dependent, care should be taken when exploiting them to intentionally alter cellular function via their ligands. Collectively, the different types of Fabps are members of a systemic network that works to coordinate metabolic functions occurring in different organs to optimize metabolism at the organismal level. Their importance extends beyond nutrient and energy metabolism and includes fine control of cellular processes involved in many aspects of organismal physiology.

## AUTHOR CONTRIBUTIONS

**Luis Agellon:** Conceptualization (lead); funding acquisition (lead); project administration (lead); writing – original draft (lead); writing – review and editing (lead).

## FUNDING INFORMATION

Our research on Fabps received funding from the Canadian Institutes of Health Research, Canada Research Chairs Program, Canada Foundation of Innovation, Natural Sciences and Engineering Research Council of Canada, and an operating grant from Ciba‐Geigy. L.B.A. held the Canada Research Chair (Tier 1) in the Biochemistry and Molecular Biology of Nutrition.

## CONFLICT OF INTEREST STATEMENT

The author has no conflict of interest to declare.

## Supporting information


Table S1.

## Data Availability

Data sharing is not applicable as no new experimental data were created or analyzed in the preparation of this article.
