# Supplementary material for: Importance of fatty acid binding proteins in cellular function and organismal metabolism
Source: J Cell Mol Med. 2023 Mar 6;28(5):e17703. doi: 10.1111/jcmm.17703 (PMC10902576; doi:10.1111/jcmm.17703)
Supplement: Supplementary file 1 — Table S1. [file JCMM-28-e17703-s001.docx]

**TABLE S1**

Tissue distribution of human FABPs

| Fabp type | Tissue distribution^a^ |
| --- | --- |
| FABP1 | <https://www.proteinatlas.org/ENSG00000163586-FABP1/tissue> |
| FABP2 | <https://www.proteinatlas.org/ENSG00000145384-FABP2/tissue> |
| FABP3 | <https://www.proteinatlas.org/ENSG00000121769-FABP3/tissue> |
| FABP4 | <https://www.proteinatlas.org/ENSG00000170323-FABP4/tissue> |
| FABP5 | <https://www.proteinatlas.org/ENSG00000164687-FABP5/tissue> |
| FABP6 | <https://www.proteinatlas.org/ENSG00000170231-FABP6/tissue> |
| FABP7 | <https://www.proteinatlas.org/ENSG00000164434-FABP7/tissue> |
| FABP8 | <https://www.proteinatlas.org/ENSG00000147588-PMP2/tissue> |
| FABP9 | <https://www.proteinatlas.org/ENSG00000205186-FABP9/tissue> |
| FABP12 | <https://www.proteinatlas.org/ENSG00000197416-FABP12/tissue> |

^a^All the URLs shown were accessed on 2022-10
